# Milk Composition of Asian Elephants (*Elephas maximus*) in a Natural Environment in Myanmar during Late Lactation

**DOI:** 10.3390/ani10040725

**Published:** 2020-04-22

**Authors:** Ellen S. Dierenfeld, Yadana A. M. Han, Khyne U. Mar, Aung Aung, Aung Thura Soe, Virpi Lummaa, Mirkka Lahdenperä

**Affiliations:** 1 Ellen S. Dierenfeld, LLC, St. Louis, MO 63128, USA; 2 School of Animal, Rural and Environmental Sciences, Nottingham Trent University, Southwell NG25 0QF, UK; 3 Department of Medical Research (Pyin-Oo-Lwin Branch), Pyin-Oo-Lwin 05081, Myanmar; yadanaamh2017@gmail.com; 4 Department of Physiology and Biochemistry, University of Veterinary Science, Yezin 05282, Myanmar; aung.a.aung@gmail.com; 5 Veterinarians International, One Penn Plaza, Suite 6337, New York, NY 10119, USA; emaximus2014@gmail.com; 6 Department of Animal and Plant Sciences, University of Sheffield, Western Bank, Sheffield S10 2TN, UK; 7 Extraction Department, Magway Region, Myanma Timber Enterprise, Magwe 0411, Myanmar; thurasoemteelephant@gmail.com; 8 Department of Biology, University of Turku, 20014 Turku, Finland; virpi.lummaa@gmail.com; 9 Department of Public Health, University of Turku and Turku University Hospital, 20520 Turku, Finland; mirkka.lahdenpera@utu.fi

**Keywords:** elephant, lactation, milk composition, nutrition, pachyderm

## Abstract

**Simple Summary:**

In this study, we analyzed longitudinal milk samples and consumed plant species from six Asian elephants managed in their natural environment and consuming native plants in Myanmar to evaluate seasonal or animal-related changes in milk content and diet. Milk from mothers nursing calves aged one-and-a-half to three years was high in fat; milk solids and protein percentages increased for older calves, and protein levels increased in both milk and plants during the wet versus dry season. Higher protein levels in plants eaten by these elephants during the wet season compared to the dry season may underlie the seasonal milk changes observed. Milk consumed by female calves was higher in protein compared with male calves. Maternal traits such as size, age, origin (captive-born vs. wild-born) and number of previous calves, were also significantly associated with milk composition. Understanding such factors influencing milk production and composition contributes to improving feeding management strategies to optimize the nutrition, health, and feeding management of both wild and captive elephant populations.

**Abstract:**

The nutritional content of milk from free-living Asian elephants has not previously been reported, despite being vital for better management of captive populations. This study analyzed both milk composition and consumed plant species of Asian elephants managed in their natural environment in Myanmar. Longitudinal samples (*n* = 36) were obtained during both the wet and the dry season from six mature females in mid to late lactation in 2016 and 2017. Milk composition averaged 82.44% water, with 17.56% total solids containing 5.23% protein, 15.10% fat, 0.87% ash, and 0.18 µg/mL vitamin E. Solids and protein increased with lactation month. Total protein in milk was higher during the wet vs. the dry season. Observed factors linked with maternal (age, parity, size and origin) and calf traits (sex) had significant associations with milk nutrient levels. Primary forages consumed contained moderate protein and fiber. Higher dietary protein during the wet season (11–25%) compared to the dry season (6–19%) may be linked with increased milk protein observed. Our results call for further field studies of milk and diet composition, over entire seasons/lactation periods, and across maternal and calf traits, to improve feeding management, with an overall goal of maximized health and survival.

## 1. Introduction

The Asian elephant (*Elephas maximus*) represents one of the most seriously endangered species of large mammals in the world [1]. One-quarter (24–29%) of all remaining Asian elephants now live in captivity in range countries in Asia and a further 1000 in zoos around the world [2,3]. With the wild populations rapidly declining worldwide [4], conservation efforts should focus on improving the survival rates of both wild and captive populations. For example, the Union of Myanmar possesses the largest semi-captive Asian elephant population globally and has an extensive history of maintaining this endangered species [5,6], with the Myanma Timber Enterprise (MTE) managing 3000+ semi-captive Asian elephants, located throughout the country in various timber extraction regions [7]. Female elephants are often managed separately from the working herd(s) in maternity camps during late pregnancy and throughout lactation, allowing training (i.e., blood or milk collection), observation, and research opportunities that target improved management, health, and calf survival [8]. The calf mortality in Myanmar is lower than in zoo populations of Asian elephants [9], but still 25.6% of calves die before reaching five years of age [10]. One in four of the calf deaths are due to maternal agalactia (lack of milk) and/or general weakness of the new-born calf [10]. Similarly, on average 19% of calves die before five years of age in wild populations of African elephants (*Loxodonta africana*) [11] and a portion of the calf deaths are due to mothers struggling to meet the lactation demands. During dry years mothers are unable to sustain milk production at a level that meets the metabolic requirements of their larger sons, and as result male calves are more likely to die [12]. Furthermore, during poor seasons or years, mothers of calves under two years of age are at greater risk of mortality as a consequence of lactation costs [11,13]. In zoos, the calf mortalities are high, especially in Asian elephants (on average 30% during the first year), and one of the most important reasons is rejection of the calf by the mother, resulting in a need for human intervention to supplementally feed the calves [14]. Many of the reasons underlying such high calf mortality are relatively well studied in elephants [9,10,11,15,16,17,18], but milk composition has gained less attention even though it may be an important factor underlying mortality. Such information, particularly from animals managed in their natural habitat, could have major potential to bring about important advances for improving calf survival and maternal well-being in diverse elephant populations. 

The elephant lactation period lasts anywhere from 2 to 8 years, and weaning is usually contingent upon when the female gives birth again [19]. For the MTE elephants, the lactation period lasts up to 4 years before calf training (taming) is initiated [16]. Osthoff et al. [20] identified four phases in the lactation period of elephants: (1) the very early (colostrum) phase occurs during the first few days of lactation; (2) early lactation—up to 12 months when large variations in milk nutrient composition are observed; (3) the transition phase between 12 and 18 months when protein and fat content increases and carbohydrate content decreases; and (4) the final (late) lactation phase from 18 months onwards when the nutrient content stabilizes but protein remains high. Unique aspects of elephant milk have been described, including small sized and highly saturated lipid globules [21,22], and the presence of elevated concentrations of lactose-derived oligosaccharides [23], as well as high levels of glucosamine [24]. Nonetheless, only limited data exist concerning milk nutrient composition of captive elephants [23,25,26,27,28], with almost none from animals in a native habitat [21,22].

Early lactation milk in both species of elephants has been shown to contain 10% to 13% solids, as compared to 17–21% solids in later stages of lactation [21,26,27,28], thus supplying essential early hydration to the calf, with mothers possibly conserving water in later lactation. Fat, protein, limited minerals (Ca, P), and energy content of Asian elephant milk have been shown to increase with stage of lactation, whereas sugar concentrations decreased [28]. However, while lactation stage differences have been described in elephants [20,28], very few longitudinal studies have been published, and results vary between females at the same institution, as well as across lactations of the same individual.

Furthermore, nutrition can have a direct impact on milk composition; this has been clearly demonstrated in livestock species. The effect on milk production and nutrient composition is variable in relation to changes in types of forage, total amounts consumed, and sources of supplementations [29,30,31]. Fat content appears most sensitive to dietary changes and can vary over a range of nearly 3.0% in cattle [32], but diet has also been shown to influence milk protein concentrations in both cattle and horses [33,34]. Therefore, variation in lactation associated with seasonality, geographic location, and vegetation/diet in both African and Asian elephants requires comprehensive longitudinal investigation. Moreover, variation in milk production associated with maternal or calf traits [35], such as maternal age, origin (wild- vs. captive-born), parity, and calf sex, have been not studied extensively in elephants, nor have the physiological effects of environment (i.e., temperature, rainfall). Quantification of dietary adequacy, as well as nutritional status of both adult (lactating females) and elephant calves, through assessment of dietary ingredients and milk composition, respectively, can help us to better understand and improve the feeding management of animals, with an overall goal of maximized health and survival. Knowledge gained may further benefit captive elephant populations around the world by providing information for the development of appropriate hand-rearing protocols for orphaned or abandoned calves [19,27,28]. 

This study was conducted to examine longitudinal variation in the composition of Asian elephant milk during the late lactation period, utilizing elephants maintained in a natural environment. Associations with milk composition were investigated in relation to climatic season in Myanmar [36,37], lactation month (=calf age), mother’s age at calf birth [38], calf sex and parity [10], mother’s origin (wild-born, captive-born; [39,40]) and size (smaller or bigger than age- and sex-specific means from the same population [41]). To evaluate variation in natural diet consumed by the elephants, we also investigated samples of 10 preferred plants eaten by the elephants, and measured the levels of crude protein, neutral detergent fiber and acid detergent fiber content during dry and wet seasons. The results should be interpreted cautiously as the sample size is small, but this study can still provide new insights and directions for further studies in milk composition, as well as potential tools for improving population management and, ultimately, maternal and calf health in diverse Asian elephant populations managed across the world. 

## 2. Methods

### 2.1. Study Population, Animals and Sample Collection

Our study focused on working elephants employed by a state-run timber extraction agency, the Myanma Timber Enterprise (MTE) in the timber logging industry. The MTE elephants live in their native forest habitat distributed across the country [42]. The elephants are used during the day as riding, transport, and draught animals, following strict set working hours, working days per year, and tonnage per individual. At night and during rest periods, all elephants forage in the forest unsupervised. Breeding rates are natural and not managed by humans with many captive-born calves thought to be sired by wild bulls, and calves born in captivity are cared for by their biological and allo-mothers [17,43]. Elephants can theoretically reproduce throughout the year; previous study in this same population shows that about 40% of births occur between December and March, corresponding to the cool, dry period [44]. Once a pregnancy is noted, females are given rest with ad libitum foraging time, along with any pair-bonded allo-mother. If the MTE has more than five pregnant females in close proximity, a separate unit is often formed that becomes a maternity camp, managed by a head-mahout or caretaker (sin-gaung). Female elephants with young calves are never separated from their offspring at the camps; females are allowed to forage freely, and calves suckle freely until natural weaning or taming at four years of age [16]. Those mothers with calves over one to two years of age are used as transport (baggage) animals in the day time, but given unsupervised foraging for 10–14 h at night, with their calves following at heel allowed access to suckling on demand. 

Six lactating female elephants ranging from 23 to 40 years of age and located at the Nat-pauk elephant camp, West Katha Timber Extraction Area, were used in this study. As is common for almost all working elephants in Myanmar (see above), these animals relied primarily on natural foraging, with limited additional dietary supplementation (detailed records unavailable). Adequate water was available ad libitum at all times. Information concerning individual animals is found in Table 1. 

#### 2.1.1. Milk

Solids components in milk (inversely related to water content and critical in maintenance of hydration status) include nutrients such as fat (provides dietary energy, essential fatty acids for cell membrane stability, as well as aiding in the absorption/metabolism of fat-soluble vitamins), protein (comprises amino acids for muscle development/growth), and ash (mineral constituents that contribute to skeletal development, as well as metabolic co-factors critical for overall health). Vitamin E was targeted as antioxidant health issues have been previously linked with low vitamin E status in captive elephants [45]. To assess variation in these components, milk samples were collected on the same day once per month (*n* = 6 per female) in July–September 2016 (wet season) and from December 2016 until February 2017 (cold, dry season), representing mid to late lactation in all females. About 10 mL of milk was collected via manual stimulation on the opposite teat during calf suckling bouts; initial secretions were not collected and targeted samples were taken mid-suckling. Collected samples were divided into two 5 mL aliquots for separate studies, transferred into labeled polyurethane vials and transported on ice within 24 h of collection. All vials were stored at −20 °C and kept frozen for up to nine months prior to batch analysis for either proximate analysis or vitamin E detection.

#### 2.1.2. Forage 

Preferred natural/local plants (*n* = 10 spp.) eaten by the study elephants were surveyed and identified by mahouts. Given the elephants forage freely at night in the forests, it was beyond the scope of this study to quantify individual-level variation in consumption or quantities. Fifty to 200 g per species was collected from multiple plants during the wet season (July through September) as well as the cold, dry season (December through February). Details of scientific and local names, plant form and parts eaten/analyzed are found in Table 2. Samples were placed into plastic bags and held in a cool box for ~24 h during transportation to the analytical laboratory, where they were immediately dried and stored until analysis (see below). 

### 2.2. Laboratory Methods

#### 2.2.1. Milk

All samples were transported to the Nutrition Laboratory in the Department of Physiology and Biochemistry, University of Veterinary Science, Yezin, Myanmar. Total solids in milk were determined using method 925.23 [46]; milk ash was determined using the gravimetric method 945.46 [46]. Milk crude protein (CP) was determined using the Kjeldahl method (990.03; Foss 2020 digester and Foss 2100 Kjeltec distillation unit, Foss Analytical AB, Höganäs, Sweden) with a copper catalyst, and using a milk-specific conversion factor for nitrogen to CP of 6.38. Crude fat in milk samples was determined according to a published low technology method [47], using separation in capillary tubes and an Adams Autocrit Centrifuge model CT-2905 (Clay-Adams, Inc., New York, NY, USA). Percentage fat in the total column was measured to 0.01 mm using digital calipers (Powerfix model Z22855, Paget Trading Ltd., London, UK), and differentiated into “liquid” fat compared to “cream” layers [8]. Milk samples were extracted with hexane, and vitamin E content determined according to the method of Dierenfeld and Dolensek [45], using HPLC (PDA plus detector, C-18 column, CT-06484, Perkin Elmer, USA) for 12 min at the wavelength of 292 nm, measured against an α-tocopherol standard for quantification. Water was calculated as 100% minus solids. Sugar was not analyzed due to limitations of laboratory equipment and reagents.

#### 2.2.2. Forage

Plant samples were dried at 100 °C to determine absolute dry matter (DM), and ashed at 550 °C to determine organic content (methods 930.15 and 942.05, respectively [46]). Determination of crude protein followed the Kjeldahl method (990.03 [46], using a Foss 2020 digester and Foss 2100 Kjeltec distillation unit) for dried forage, with a 6.25 conversion factor; neutral detergent fiber (NDF) and acid detergent fiber (ADF), were analyzed by the methods of Goering and Van Soest [48]. NDF comprises hemicellulose (partially digestible as well as fermentable fiber), cellulose (fermentable fiber), and lignin (non-available dietary fiber) fractions, whereas ADF comprises only cellulose and lignin—both latter components contribute physical dietary fiber bulk, but only cellulose (in ADF) can be fermented by gut microbes to supply potential energy to herbivores [48]. All forage values are reported throughout on a DM basis. 

### 2.3. Statistical Analyses

#### 2.3.1. Milk

The statistical analyses of the milk composition (*n* = 36) were conducted with general linear models (GLMs) in SAS (SAS Institute Inc., Cary, NC, USA, release 9.4, 2014). However, to minimize pseudo-replication due to repeated measures on the same mothers, we also conducted the analyses by including the within-mother variation using inverse-variance weighting models [49]. These models, as well as more robust generalized estimating equation (GEE) models with maternal ID as a repeated term, produced similar results as the final models reported in this article and confirmed our conclusions. The residuals of all models were normally distributed and the variances were equal. Statistical significance was set at *p* = 0.05. Outliers were detected with studentized residuals (>±3) and as a result one value for fat percentage was removed from the analysis (total *n*(fat) = 35). The same variables were controlled for in the analyses of all milk components. Lactation month (=calf age in months, mean 25.0 ± 5.57, range 17–38) was retained in the final analyses, despite not always being significant, due to previously reported strong associations with milk components [28]. Although we sampled slightly different lactation months for male calves (17 to 38 month) compared with females (17 to 26 month of age), long periods of age overlap were observed, and results were the same when lactation month was not included in the final model when non-significant. The other controlled variables were the continuous term of mother’s age at calf birth (mean 31.17 ± 6.99, range 22–40), categorized parity (1 = 2nd born (*n* = 18); 2 = 4th or 5th born (*n* = 18)), calf sex (M = male (*n* = 24); F = female (*n* = 12)), season at the time of collection (July 2016, Aug 2016, Sep 2016 = wet (*n* = 18); December 2016, January 2017, March 2017 = dry (*n* = 18)), mother’s origin (wild-born (*n* = 12 samples); captive-born (*n* = 24) and mother’s size measured at 31 December 2015 by EziWeigh 3000 weighing scale (smaller (*n* = 12)/bigger (*n* = 24) than same-aged females in the population, estimated from the age- and sex-specific weight curve from the population [41]). 

#### 2.3.2. Forage

Paired *t*-comparisons of means were analyzed during the wet vs. the cold, dry seasons for chemical composition (crude protein, neutral detergent fiber, acid detergent fiber, ash) for forage samples of 10 species (Table 2), and descriptive statistics (mean ± standard deviation (SD) were conducted using SPSS (Version 20.0, IBM Corp., Armonk, NY, USA), with statistical significance set at *p* = 0.05.

## 3. Results 

### 3.1. Milk

Milk composition over the eight-month collection period averaged 82.44% water, with 17.56% total solids containing 5.23% protein, 15.10% fat, 0.87% ash, and 0.18 µg/mL vitamin E (Table 3). Lipid layers in the milk samples were variable, but primarily liquid (~80%) and clear in color; conversely, the opaque cream layer made up only ~20% of total milk fat quantified (Table 3). 

**Figure 1:**
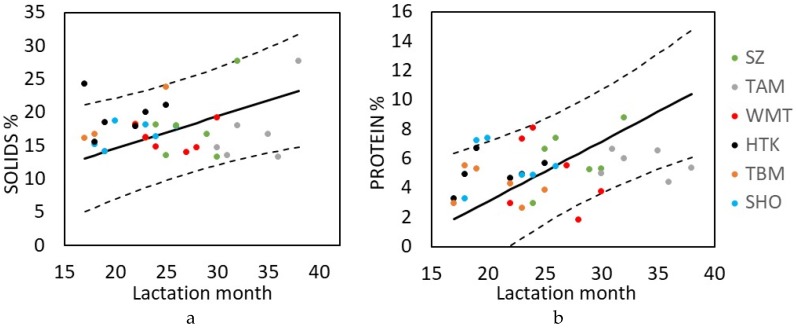
Variation in milk components with lactation month (calf age). Solids (**a**) and protein components (**b**) increased in later lactation months during late lactation in females’ milk. Points represent raw data for different mothers and solid lines show predicted values from the model with CLs (dashed lines). Predicted values drawn from the model according to reference categories and captive-born mothers. SZ, TAM, WMT, HTK, TBM, SHO: Elephant names coded as initials of house names.

Lactation month, i.e., calf age, is known to associate with Asian elephant milk composition, at least in zoo settings [26,28]. In the milk of the timber elephants, solids content and one component in it, protein, were positively correlated with lactation month (*β* = 0.41 ± 0.13, *p* = 0.003; *β* = 0.48 ± 0.19, *p* = 0.016, respectively), increasing in later lactation months (Figure 1, Tables S1–S3). 

**Figure 2:**
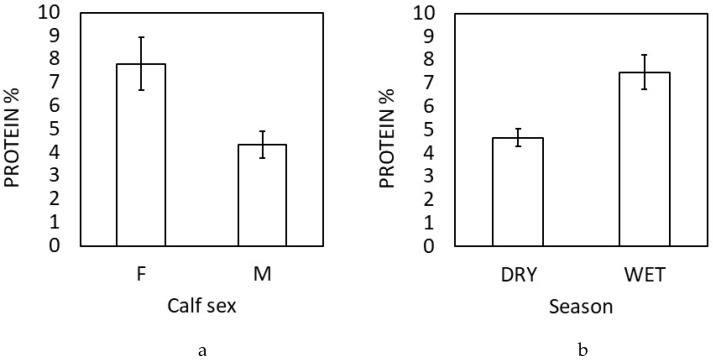
Sex and seasonal differences in protein composition of elephant milk. Protein composition was higher in milk of mothers with female calves compared to male calves (**a**) and protein content in milk was higher during the wet season compared to the cold, dry season in Myanmar (**b**). Figures show standard errors (error bars) and predicted means (bars).

Calf sex was significantly associated with the protein content in milk (*p* = 0.039, Table S1); mothers of male calves displayed 44.4% lower protein content than mothers of female calves (Figure 2a). Protein content also differed significantly in milk samples from the same elephants collected in the cold, dry season compared to the wet season (*β* = −2.81 ± 0.84, *p* = 0.002), being 60.2% higher during the wet season from July to September than during the cold, dry season from December 2016 to March 2017 (Figure 2b, Table S1). There were no seasonal differences noted on other milk components (Tables S1–S6).

**Figure 3:**
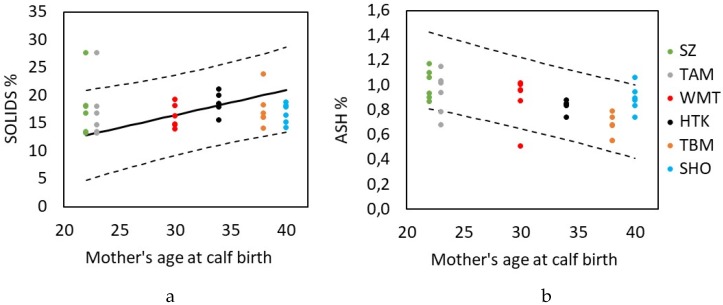
Variation in milk components with maternal age at calf birth. Solids components (**a**) increased with greater maternal age whereas (**b**) ash components in late lactation milk. Points represent raw data for different mothers and solid lines show predicted values from the model with CLs (dashed lines). Predicted values drawn from the model according to reference categories and captive-born mothers.

Maternal traits showed significant association with the variation in different milk components. Maternal age at calf birth had a significant association with the solids and ash components in the milk. Solids content increased 62.9% (*β* = 0.45 ± 0.18, *p* = 0.015, Figure 3a, Table S2) and ash content decreased 36.8% from a 22-year-old mother to a 40-year-old mother (*β* = −0.023 ± 0.005, *p* = 0.0002, Figure 3b, Table S5). 

Wild-born mothers had 21% lower solids content (wild-born: 15.40 ± 0.73 vs. captive-born: 18.64 ± 0.73, *p* = 0.028, Table S2), and 14.1% higher ash content (wild-born: 0.97 ± 0.042 vs. captive-born: 0.85 ± 0.030, *p* = 0.027, Table S5) in milk compared to captive-born mothers. Smaller mothers (in relation to age- and sex-specific population weight curves) had 31.4% higher levels of protein (smaller: 6.87 ± 0.38 vs. larger: 5.23 ± 0.38, *p* = 0.025, Table S1) and 14.1% higher ash content (smaller: 0.97 ± 0.045 vs. larger: 0.85 ± 0.031, *p* = 0.044, Table S5) in milk than larger mothers. Finally, protein, solids, water, and vitamin E content in milk (Tables S1–S3, S6) varied with mother’s parity. Protein and solids content were 37.7% and 17.6% lower for mothers with later parities (4, 5: 4.65 ± 0.45 and 15.38 ± 1.03, respectively) than earlier parity (2: 7.48 ± 0.95 and 18.66 ± 0.91, respectively) (*p* = 0.031, Table S1; *p* = 0.037, Table S2, respectively). On the contrary, water and vitamin content were 4.0% and 4.9% increased for later parities compared to earlier parity (2: 81.34 ± 0.91 vs. 4,5: 84.62 ± 1.03, *p* = 0.037, Table S3; 2: 0.17 ± 0.0029 vs. 4,5: 0.18 ± 0.0030, *p* = 0.049, Table S6, respectively). Fat composition of milk (Table S4) showed no change in association with any of the variables examined in this study.

### 3.2. Forage

**Figure 4:**
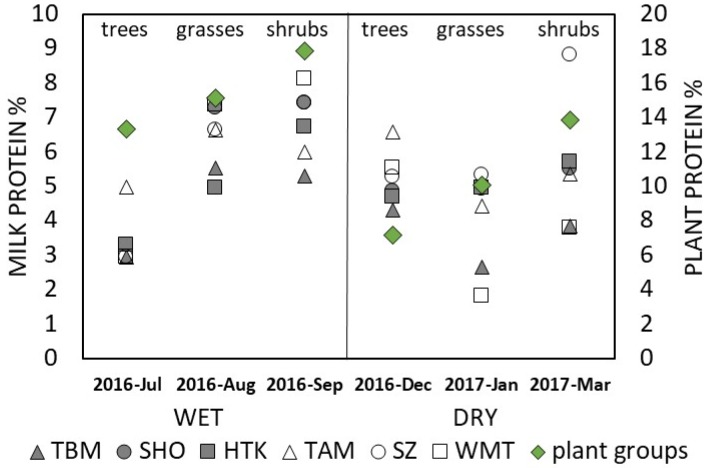
Seasonal changes in milk protein vs. forage protein by three plant groups. Milk and forage protein values were higher during wet season compared to dry season values (*n* = 36 milk protein values from six mothers, mothers identified by different symbols; *n* = 6 mean forage protein values (green symbols) of three different plant groups (trees, grasses, shrubs, according to Table 4).

Primary food plants consumed by the elephants in different seasons were analyzed for select nutrients and contained 13.43% ± 5.04% crude protein, 51.64% ± 14.86% neutral detergent fiber, 37.65% ± 7.30% acid detergent fiber, and 14.76% ± 4.85% ash. Seasonal chemical composition of forages is displayed in Table 4. The measured crude protein was significantly (*t*-value = 3.534, *p* = 0.003) higher in wet versus dry season plants, but other constituents did not vary seasonally (paired comparisons for NDF, ADF, and ash were all *p* > 0.05). Seasonal differences in forage protein and milk protein values are displayed in Figure 4. 

## 4. Discussion

This study analyzed milk composition and forage consumed by Asian elephants in Myanmar. We report the first values of five milk components for females foraging in a free-range setting in their native habitat: solids (inverse of water and comprising fat, protein, ash) and vitamin E. We found that these milk components varied with the lactation month, season, and several maternal and calf traits. We also found that the protein content of forage consumed by the elephants varied with the season, possibly associated with demonstrated protein changes in milk across seasons. The first results of milk composition of Asian elephants living in their natural habitat reported here urge for more studies, with greater sample sizes to identify individuals in need of supplementation, as well as to further identify various factors associated with milk composition to increase maternal and calf nutrition, wellbeing, and survival in different elephant populations. 

Percentages of total solids, protein, and fat in elephant milk have been reported to vary throughout lactation, and tend to increase over the lactation period [20,21,28]. In the current study, milk samples were collected from females almost exclusively (34/36; 94% of samples) during the final lactation phase (calf ages ≥18 months). Values for total solids in our milk samples (17.6%) are similar to those previously reported at post-early phase for Asian elephants kept in zoo conditions (17.3%, 19.7% ± 2.7%, 19.6% ± 1.2%; [25,26,28], respectively). Therefore our results suggest that total solids content of elephant milk can increase for longer than previously reported (until 38 months in this study), but the earlier solids contents may have been at lower levels in MTE elephants than zoo elephants and was not measured in this study. 

Total protein content of African elephant milk ranges between 2.2% and 6.4%, and that of the Asian elephant between 3.4% and 6.5%, depending on the lactation stage [20]. In this study, protein values in milk from MTE elephants (5.23%) varied seasonally and were higher than values previously reported for zoo elephants (3% to 4%) in early to mid- lactation [26,27,28], but within ranges previously reported for Asian elephants in late lactation [28]. The protein content of milk samples collected during the wet season was higher by almost 3% compared with samples from the cold, dry season, possibly linked to climate and diet. However, due to small sample size and differences in milk vs. forage sample collection, we were not able to statistically test this relationship directly. Crude protein content of plants increases over the rainy season [50], and we also found significantly higher protein values in elephant fodder measured in this study during the wet season. Although increasing dietary crude protein had little effect on milk protein percentages in dairy cattle [51], increased dietary protein [52], as well as forage (and fiber) intake resulted in higher protein content in mare’s milk [34]. The impacts of seasonal variation on the nutritional value of herbage and milk composition have not been previously documented in elephants. Analyzed (this study) and calculated diets (zoo diets; [26,28,53]) suggest a range of ~9–14% crude protein associated with the observed milk compositions, supporting recommendations for reproduction and lactation in elephants [53].

Fat content of African elephant milk ranges from 1.7% to 17%, and that of the Asian elephant between 0.63% and 19%, depending on lactation phase [20,28,54]. Total fat content in milk from Myanma elephants was considerably higher (15.19% ± 3.87%) compared to previously reported values of 9.1% to 13.2% (up to about seven months; [54], or 7.6% ± 2.6% to 8.3% ± 1.0% in early lactation (up to about nine months; [26,28], but almost identical to previously reported values for late lactation (15% to 18% at 18 to 30 months [28]). Differences in fat content across various published studies are likely due to stage of lactation sampled, although yield and diet (and seasonality) may also have impact [8]. Milk fat content is increased by high forage feeding in other herbivores, including dairy cattle [55,56] and mares [34], and may have the same effect on the elephants’ total milk lipids. Details of diet, however, have been documented only in few studies associated with elephant milk nutrient composition and only with captive-managed animals (i.e., [26,28]). Although we saw no seasonal association of diet with milk fat content in this study, ranges of dietary fiber were similar to those calculated from diets associated with milk composition in zoo studies (approximately NDF 55–65%, and ADF 30–40% (DM basis) [26,28], and fiber is known to associate with milk fat in other species [32,55].

The variable fat fractions (liquid vs. cream layers) observed in our samples are particularly noteworthy: McCullagh and Widdowson [21] previously reported that African elephant milk lipid globules are half the size of bovine milk fats, with a unique fatty acid signature; fatty acid details of Asian elephant milk have not yet been characterized in detail, apart from a single published account from a zoo individual [54]. Osthoff et al. [22] confirmed a high content (~70%) of short-chain saturated fatty acids (FAs), particularly capric and lauric acids, low levels of polyunsaturated FAs, and omega-3:omega-6 FA ratios of approximately 1:1 in mid-lactation African elephant milk, with an increasing degree of short chain FAs as lactation progresses. This same pattern was seen in Asian elephant milk from samples taken at days 24, 103, and 154 of lactation, with up to ~80% short-chain FAs [54]. Regulatory mechanisms for this process are not yet defined, nor have they been examined in Asian elephant milk. While smaller and shorter chain lipid molecules may be more liquid at room temperatures, the observation of widely ranging proportions of both liquid and cream fat fractions in the centrifuged elephant milk samples documented in this study need to be investigated further. It is tempting to speculate that the highest proportion of liquid lipid fractions, found in samples from the two elephant cows with the oldest calves, may represent this pattern of increased short chain FAs in later lactation milk, but no clear pattern of variation (by individual, lactation month, or season) was discernible in our limited data set. Further detailed analyses of specific fatty acids are required to characterize these separate milk fractions. 

Overall, the ash content of elephant milk in this study (~0.9% ± 0.2%) remained constant throughout the collection period, but an association with female traits including age (decreased in older mothers), origin, and size (higher in wild-born and smaller animals) was suggested. Because milk mineral content is influenced by mastitis in dairy cattle [57], it might be useful to consider multiple health aspects of females, and possible impacts on calf nutrition/health, when assessing milk quality in elephants. Average ash values were somewhat higher in Myanmar elephant milk samples compared to previous literature reports (0.54% ± 0.03%, [26]; 0.5% to 0.8% for Asian elephants and 0.73% ± 0.02% for African elephants, [20]). In a long-term study (three years), Abbondanza et al. [28] reported that there were strong positive correlations among Asian elephant milk constituents (fat, protein, and ash) and calf age. In that respect, it would be valuable to more thoroughly investigate specific minerals that are required as essential nutrients for skeletal growth and cellular metabolism in Myanma elephant milk samples, as well as vegetation, particularly relevant to overall mineral nutrition of cows and calves [53]. 

Vitamin E content of milk in this study, measured as α-tocopherol, averaged 0.18 ± 0.01 µg/mL, and did not vary seasonally. The only other published data on Asian elephant milk vitamin E content recorded a range of 0.09 to 0.55 µg/mL in a zoo animal, with an average value of 0.33 ± 0.12 µg/mL during the first 280 days of lactation [26]. The lower vitamin E concentrations in milk samples from the current study may be due to sampling or analytical issues, reduced vitamin E status, higher overall milk fat content, and/or a high proportion of polyunsaturated fatty acids in Myanma elephant milk that may oxidize vitamin E. Notably, the concentration ranges for vitamin E in milk samples from both studies are similar to those found in plasma in Asian elephants [45] and unpublished data (from both African and Asian elephants, Dierenfeld), suggesting a strong correlation between milk and plasma concentrations (reflecting status) of this nutrient. Further detailed investigations, including assessment of dietary vitamin E levels and vitamin E status of cows and/or calves, as well as changes in this nutrient over the lactation period, would help clarify metabolism of this critical antioxidant nutrient [45] in elephants. 

Milk components were also found to differ with specific maternal and calf traits (mother age, parity, origin, size, and calf sex) which have not previously been examined in Asian elephants. This is primarily due to limitations in sample sizes, yet significant relationships were detected even within our limited data set. In particular, the statistically significant association between calf sex and milk protein concentration may be of note. Samples from male calves were associated with later months of lactation, and protein content has been shown to increase later in lactation (Figure 1) [20,26,28]. Since lactation month was controlled in the analysis, the observed sex association with protein appears to be separate from the positive correlation of lactation month on protein level. Lower milk protein content from cows nursing male calves, at a later stage of lactation, is opposite to what might be expected, and contrary to the pattern reported in a variety of other mammals biased toward male offspring [58,59,60,61], Thus the sex difference may be of biological significance. Abbondanza et al. [28] also report significant differences in milk composition from the same female elephant over two lactations with different-sexed calves in a zoo setting, but specific details of those nutrient differences were not provided. Anecdotally, a milk replacer fed to a male Asian elephant calf, formulated based on analysis of milk samples obtained from an elephant cow nursing a female calf at the same institution, required multiple adjustments of fat and energy content to produce optimal intake and growth responses [54]. Hinde et al. [35] reported no compositional differences between milks from Holstein cattle birthing male vs. female calves (fat 3.6%, protein 3.2%), but found total volume produced was correlated with calf sex. It may be that milk yield also differs between the mothers of male and female elephant calves; sex-influenced suckling demands [12] and growth rates [41] may impact milk composition (or vice versa) but these aspects remain to be investigated in elephants. Resource allocation theory suggests that maternal investment should be biased toward the sex that gains more reproductive advantage [61]. In a long-lived species with a matriarchal society such as the elephant, with higher survival for females [39], higher milk protein provided to females to support growth and survival may be a contrasting advantageous strategy. 

We found mixed relationship patterns between maternal age and parity on milk composition. Several studies in humans have shown that primiparous and younger women have higher concentrations of several constituents in milk and women with very high parities produce milk of reduced quality [62]. Similar patterns are found in dairy cattle, goats, and camels, where increasing age and parity are typically correlated with lower milk solids (cattle) and lower protein and fat content [63,64,65]. Milk quality in dairy ewes and mares, however, increased with parity [66,67], which has been attributed to udder development and better energy balance. In the milk of elephants, we found that solids and protein content decreased in later parities. However, we also found that solids content increased the older the mother. These results are especially interesting, as it is known in this population that first-born calves and calves born to older mothers have lower survival probabilities to five years of age [10]. However, the age effect is known to be restricted to captive-born mothers only [38], whereas this study included both captive-born and wild-born mothers. Also we did not have any first-born calves in this study or very young or old mothers (<20 or >50 years), preventing us from observing the effects of these potentially adverse conditions on milk composition. Nonetheless, this study with medium range mother ages (22–40 years) and parities (2 vs. 4/5), suggests that older mothers, at least up to a point, might actually produce milk of higher quality, at least compared to the milk of mothers at peak reproductive ages, but this may depend on their parity. Furthermore, we also found that wild-born mothers had lower solids content in milk than captive-born mothers. Wild-born mothers have higher mortality, lower reproductive rates, and lower calf survival in Myanmar [39,40], potentially due to increased stress of capture, and may thus also produce milk of lower quality. Finally, we also observed smaller mothers producing milk with higher protein levels. However, all mothers in this study were in good condition and the differences in size were relatively small. In addition, we only had one weight measure per mother whereas longitudinal weight measures or body scores would produce much more detailed information on a mother’s ability to produce milk as their calf grows. To investigate in more detail the link between observed calf mortality patterns and milk composition, we would need a larger sample size of mothers measured longitudinally in different contexts, as well as studies of milk yield (which can compensate milk quality) to also clarify why some mothers suffer from milk agalactia (lack of milk after parturition), associated with many calf mortalities in Myanmar [10]. 

Forage composition and animal behavior may further associate with milk composition. Although seasonal differences in the diversity and selectivity of food choices by elephants in Myanmar have been previously reported [68,69], detailed nutrient analyses have not been conducted. While the composition of some native plants eaten by the MTE elephants certainly varied seasonally, others appeared less affected. The wide variation in both crude protein (6% to ~19% in the dry season, 11% to 25% in the cold, wet season) and fiber fractions (i.e., NDF 35% to 75% in the dry versus 29% to 72% in the wet season) of forages consumed could have allowed individual animals to selectively consume diets of widely differing composition during both seasons. Quantitative feeding behavioral observations were not a part of this study; Campos-Arceiz et al. [69] reported that elephants selectively consumed bark and fruits of several preferred browses—plant parts that were not examined herein. These earlier field studies further confirmed that elephants in Myanmar are primarily browsers, although bamboo (a grass) was considered a browse (due to growth form), with only seven taxa providing 97% of forage eaten in one study [68]. Nonetheless, the overall nutritional status of females can only be speculated; certainly the nutritional status was at least adequate to support reproduction and lactation in these dams. Of the 10 species analyzed, bamboos (*Cephalostachum pergracile*, *Dendrocalamus brandisii*, and *D. membranaceus*) were the favorite forages of MTE elephants from the Nat-pauk camp (mahouts, personal communication), confirming the observations of [68] where bamboo comprised 57% of intake. Overall, plants in general appeared to contain moderate to high crude protein levels, and moderate to high fiber levels with quality characteristics within recommended levels for feeding captive elephants [53], albeit many details (particularly mineral nutrition) are lacking in this current dataset. Nonetheless, the limited nutrient data provide some insights into the composition of foods that can support lactation of elephants in Myanmar. The quite high ash values recorded (6% to >20% of dry matter, averaging ~15%) in the plants consumed suggest that mineral nutrition should be investigated in more detail in these elephants; mineral nutrition of elephants (particularly free-ranging) is an area with a paucity of published data. Balanced mineral nutrition is required for optimal health, growth, and milk production in all species, and select milk mineral nutrients (selenium and iodine) can be effectively manipulated through dietary intake in dairy cattle [70]. Correlating dietary and milk mineral concentrations in lactating females could provide important insights into health and survival of elephant calves.

## 5. Conclusions

Milk composition from six elephant cows over an eight-month period of late lactation in a native environment was similar to that previously reported for three zoo-managed Asian elephants [28]. From a nutritional perspective, higher quality milk was produced during the wet season, and likely provided calves with a better health status during that time frame compared to the cold, dry season. Detailed feeding observations may provide more insight into the direct impacts of diet on milk composition in elephants. According to this study, the forages mostly consumed by the elephants were moderate to high in crude protein, and moderate to high in fiber, relative to recommended levels for feeding captive elephants. The crude protein content of forage was higher in the wet season compared with the cold, dry season, as was the milk protein content. High fiber in consumed forage throughout the year may have resulted in high milk fat, but values were within expected ranges for late-lactation milk samples and did not vary seasonally. 

The wide variability in proportions of lipid fractions found in these milk samples (liquid vs. cream) needs more detailed study, especially in relation to diet and stage of lactation. Continued longitudinal milk sampling studies with more animals to further characterize/confirm composition of early- and mid-lactation milk samples (in addition to late lactation), and changes over the lactation period, of Asian elephants in a natural environment would add to the limited literature on this topic. Quantification of vitamin E concentrations in milk, forage, and plasma samples would contribute to baseline information on nutritional status and provide guidelines for improved health status of managed global elephant populations. From a nutritional perspective, the substantial mineral (ash) fraction identified in milk and forage samples from Myanma elephants should be analyzed in more detail in relation to diets consumed, and correlated with nutritional status of both cows and calves. 

Finally, results from this study confirm the validity/value of zoo-based elephant milk samples to reflect lactation physiology in situ. While stage of lactation has been shown to substantially impact milk composition, associations with female (age, origin, parity, body condition, diet composition), and calf traits should also be recognized as important variables to consider. From these data, female calves born from the smallest, captive-bred, older second-time mothers during the wet season in Myanmar might be expected to be exposed to the highest quality milk, providing excellent growth/health potential for the calves. 

## Figures and Tables

**Table 1:** Details of Asian elephant cows sampled from the Nat-pauk elephant camp, West Katha Timber Extraction Area in Myanmar.

Name of Elephant	Age at Birth (Years)	Origin	Parity No.	Body Weight (kg)	Date of Calf Birth	Sex of Calf	Months of Lactation Sampled
SZ	22	C	2	2404 (B)	1.7.2014	M	24–32
TAM	23	C	4	2582 (B)	15.1.2014	M	30–38
WMT	30	W	2	2706 (B)	22.9.2014	M	22–30
HTK	34	C	2	2407 (S)	26.2.2015	M	17–25
TBM	38	C	5	2964 (B)	22.2.2015	F	17–25
SHO	40	W	4	2358 (S)	1.1.2015	F	18–26

SZ, TAM, WMT, HTK, TBM, SHO: Elephant names coded as initials of house names; B: bigger than the average-sized same-aged female in the population [40]; S: smaller; W: wild-born; C: captive-born; F: female; M: male.

**Table 2:** Preferred plants eaten by Asian elephants (*Elephas maximus*) at Nat-pauk maternity camp near Katha, Myanmar.

Local Name	Botanical Name	Type of Plant	Common Name	Part Eaten
Tin-War	*Cephalostachum pergracile*	Grass(Tree)	Bamboo	Leaf
Kaung-Si-Nwel	UNKNOWN	Shrub		Entire plant
Khway-Ei-Poke-Nwel	*Sarcococca pruniformis*	Shrub	Fleshy berry	Entire plant
Wabo-War	*Dendrocalamus brandisii*	Grass(Tree)	Bamboo	Leaf
Kha-Oung	*Ficus hispida*	Tree	Hairy Fig	Leaf
Pang-Zauk-Htoe	UNKNOWN	Shrub		Entire plant
Tama-saing	*Thysanolaena maxima*	Grass	Tiger Grass	Leaf
Ka-Pyin-Nwel	*Quercus fenestrata*	Tree	Oak Tree	Leaf
Dant-Kywe	*Cassia sophera*	Shrub	Senna Sophera	Leaf
Wa-Phyu-War	*Dendrocalamus membranaceus*	Grass(Tree)	Bamboo	Leaf

**Table 3:** Milk composition from six Asian elephants (*Elephas maximus*) consuming a natural diet in Myanmar sampled over an eight-month period during late lactation (17–38 months) (*n* = 36 samples). Data (mean ± standard deviation (SD)) presented on a wet basis.

Constituent	Mean ± SD	Range
Water, %	82.44 ± 3.65	(72.28–86.74)
Total Solids, %	17.56 ± 3.65	(13.26–27.72)
Total Fat, %	15.10 ± 3.87 ^1^	(7.59–23.48)
Liquid fat layer, % of total fat	79.19 ± 12.44	(38.77–95.75)
Cream fat layer, % of total fat	20.81 ± 12.44	(4.25–61.23)
Crude Protein, %	5.23 ± 1.66	(1.82–8.81)
Ash, %	0.87 ± 0.16	(0.51–1.17)
Vitamin E (µg/mL)	0.18 ± 0.014	(0.16–0.21)

^1^ One sample from the wet season was deemed an outlier and excluded from fat analyses.

**Table 4:** Chemical composition of primary natural forages consumed by Asian elephants during late lactation near Katha, Myanmar. Data presented on a dry matter basis.

Species	CP, %	CP, %	NDF, %	NDF, %	ADF, %	ADF, %	Ash, %	Ash, %
Season	Dry	Wet	Dry	Wet	Dry	Wet	Dry	Wet
Wabo-war *Dendrocalamus brandisii*	12.70	16.72	66.92	67.60	41.21	37.88	11.55	10.60
Tin-war *Cephalostachum pergracile*	8.82	11.26	59.49	67.18	46.55	39.45	21.65	16.80
Wa-phyu-war *Dendrocalamus membranaceus*	10.95	19.39	58.56	68.76	40.31	30.36	16.76	10.16
Tama-saing *Thysanolaena maxima*	7.75	13.08	74.67	72.08	45.80	38.60	10.36	11.15
Khway-ei-poke-nwel *Sarcococca pruniformis*	8.61	10.61	46.92	52.69	38.47	45.86	10.50	14.23
Ka-pyin-nwel *Quercus fenestrate*	6.03	13.18	61.35	32.27	51.21	26.60	6.14	8.67
Dant-kywe *Cassia sophera*	18.59	22.39	37.59	28.60	34.60	19.51	15.32	13.79
Kaung-si-nwel	12.06	25.34	38.50	43.03	33.48	42.36	15.67	17.09
Pan-zauk-htoe	16.21	13.03	34.79	40.29	34.94	35.65	19.90	23.26
Kha-oung *Ficus hispida*	8.35	13.54	40.74	40.80	37.75	32.43	21.53	20.05
Mean	** 11.01	15.86	51.95	51.33	40.43	34.87	14.94	14.58
±SD	3.97	4.97	13.99	16.44	5.84	7.83	5.23	4.71

** Paired seasonal comparison significantly different *p* < 0.01; Season: Dry: December 2016 through February 2017; Wet: July through September 2016; CP: crude protein, NDF: neutral detergent fiber, ADF: acid detergent fiber.

## Supplementary Materials

The following are available online at https://www.mdpi.com/2076-2615/10/4/725/s1, Table S1: GLM model of the effects of female and calf variables on protein composition in milk samples, Table S2: GLM model of the effects of female and calf variables on solids composition in milk samples, Table S3: GLMM model of the effects of female and calf variables on water composition in milk samples, Table S4: GLM model of the effects of female and calf variables on fat composition in milk samples, Table S5: GLM model of the effects of female and calf variables on ash composition in milk samples, Table S6: GLMM model of the effects of female and calf variables on vitamin E composition in milk samples.
