# Supplementary material for: Milk Composition of Asian Elephants (Elephas maximus) in a Natural Environment in Myanmar during Late Lactation
Source: Animals (Basel). 2020 Apr 22;10(4):725. doi: 10.3390/ani10040725 (PMC7222762; doi:10.3390/ani10040725)
Supplement: Supplementary file 1 [file animals-10-00725-s001.pdf]

Supplementary tables

Table S1. GLM model of the effects of female and calf variables on protein composition in milk samples (n=36). Reference categories are given in brackets and estimates for the other category. R^2=0.32

Term	Parameter estimate ± S.E	F	Df	*P* value
Lactation month (Calf age)	0.407 ± 0.127	10.31	1	0.003
Season (Wet)	-2.809 ± 0.837	11.27	1	0.002
Mother's size (Smaller)	-1.590 ± 0.676	5.53	1	0.025
Calf birth-order (Later-born (4,5))	2.824 ± 1.245	5.15	1	0.031
Calf sex (Male)	3.460 ± 1.603	4.66	1	0.039
Mother's origin (Wild)	0.569 ± 0.615	0.95	1	0.337
Maternal age at calf birth	0.014 ± 0.124	0.01	1	0.913

Terms retained in the final model are shown above the dashed line.

Table S2. GLM model of the effects of female and calf variables on solids composition in milk samples (n=36). Reference categories are given in brackets and estimates are given for the other category. R^2=0.23

Term	Parameter estimate ± S.E	F	Df	*P* value
Lactation month (Calf age)	0.484 ± 0.189	6.50	1	0.016
Maternal age at calf birth	0.454 ± 0.176	6.64	1	0.015
Mother's origin (Wild)	3.247 ± 1.404	5.35	1	0.028
Calf birth-order (Later-born (4,5))	3.282 ± 1.503	4.77	1	0.037
Season (Wet)	-3.379 ± 2.494	1.84	1	0.186
Mother's size (Smaller)	-0.941 ± 1.591	0.35	1	0.558
Calf sex (Male)	0.807 ± 3.221	0.06	1	0.804

Terms retained in the final model are shown above the dashed line.

Table S3. GLMM model of the effects of female and calf variables on water composition in milk samples (n=36). Reference categories are given in brackets and estimates for the other category. R^2=0.23

Term	Parameter estimate ± S.E	F	Df	*P* value
Lactation month (Calf age)	-0.484 ± 0.190	6.50	1	0.016
Maternal age at calf birth	-0.454 ± 0.176	6.64	1	0.015
Mother's origin (Wild)	-3.247 ± 1.404	5.35	1	0.028
Calf birth-order (Later-born (4,5))	-3.282 ± 1.503	4.77	1	0.037
Season (Wet)	3.379 ± 2.494	1.84	1	0.186
Mother's size (Smaller)	1.341 ± 1.771	0.57	1	0.455
Calf sex (Male)	-0.807 ± 3.221	0.06	1	0.804

Terms retained in the final model are shown above the dashed line.

Table S4. GLM model of the effects of female and calf variables on fat composition in milk samples (n=35). Reference categories are given in brackets and estimates for the other category. R^2=0.066

Term	Parameter estimate ± S.E	F	Df	*P* value
Lactation month (Calf age)	-0.181 ± 0.119	2.32	1	0.138
Calf birth-order (Later-born (4,5))	1.237 ± 1.287	0.92	1	0.344
Maternal age at calf birth	-0.113 ± 0.139	0.66	1	0.423
Calf sex (Male)	-1.043 ± 1.627	0.41	1	0.526
Season (Wet)	0.819 ± 1.467	0.31	1	0.581
Mother's origin (Wild)	-0.595 ± 1.401	0.18	1	0.674
Mother's size (Smaller)	-0.274 ± 1.584	0.03	1	0.864

Terms retained in the final model are shown above the dashed line.

Table S5. GLM model of the effects of female and calf variables on ash composition in milk samples (n=36). Reference categories are given in brackets and estimates for the other category for two level variables. R^2=0.42

Term	Parameter estimate ± S.E	F	Df	*P* value
Lactation month (Calf age)	-0.0065 ± 0.006	1.16	1	0.289
Maternal age at calf birth	-0.0229 ± 0.005	17.33	1	0.0002
Mother's origin (Wild)	-0.120 ± 0.051	5.41	1	0.027
Mother's size (Smaller)	-0.122 ± 0.058	4.39	1	0.044
Calf sex (Male)	0.146 ± 0.091	2.58	1	0.128
Calf birth-order (Later-born (4,5))	-0.080 ± 0.060	1.81	1	0.189
Season (Wet)	-0.009 ± 0.066	0.02	1	0.892

Terms retained in the final model are shown above the dashed line.

Table S6. GLMM model of the effects of female and calf variables on vitamin E composition in milk samples (n=36). Reference categories are given in brackets and estimates for the other category.

Term	Parameter estimate ± S.E	F	NumDf	DenDf	*P* value
Lactation month (Calf age)	0.0023 ± 0.002	1.16	1	33	0.289
Calf birth-order (Later-born (4,5))	-0.048 ± 0.024	4.16	1	33	0.049
Maternal age at calf birth	-0.0039 ± 0.0031	1.59	1	32	0.216
Mother's origin (Wild)	0.026 ± 0.025	1.12	1	32	0.298
Mother's size (Smaller)	0.0196 ± 0.0285	0.47	1	32	0.50
Calf sex (Male)	-0.027 ± 0.054	0.25	1	32	0.62
Season (Wet)	-0.0096 ± 0.0271	0.13	1	32	0.72

Terms retained in the final model are shown above the dashed line.
